# Panton-Valentine Leukocidin–positive *Staphylococcus aureus*, Singapore

**DOI:** 10.3201/eid1008.031088

**Published:** 2004-08

**Authors:** Li-Yang Hsu, Tse-Hsien Koh, Devanand Anantham, Asok Kurup, Kenneth Ping Wah Chan, Ban-Hock Tan

**Affiliations:** * Singapore General Hospital, Singapore

**Keywords:** letter, Panton-Valentine leukocidin, PVL, necrotizing pneumonia, Singapore

**To the Editor:** Necrotizing community-acquired pneumonia attributable to Panton-Valentine leukocidin–producing strains of *Staphylococcus aureus* has been described as a distinct clinical syndrome with a high death rate in young, immunocompetent patients (1,2). This letter details the first reported case of necrotizing pneumonia caused by Panton-Valentine leukocidin-positive *S. aureus* in a southeastern Asian country, Singapore.

An 18-year-old girl of Chinese ethnicity with a 4-day history of fever, cough, hemoptysis, and dyspnea sought treatment at Singapore General Hospital in October 2003. This episode had immediately followed an influenza-like prodromal illness for which a general practitioner had prescribed oral erythromycin ethinylsuccinate and medications for symptomatic relief. Her medical history showed an intrauterine *Toxoplasma gondii* infection that had resulted in developmental delay and slight mental retardation. She had never traveled outside Singapore.

On admission, the patient's temperature was 38.4°C, blood pressure was 130/70 mm Hg, and her pulse rate was 108 per min. Bibasal crackles were heard on auscultation of her lung fields, and her respiratory rate was 30 per min despite the use of supplemental oxygen. The results of physical examination were otherwise unremarkable. Initial chest x-ray showed air-space shadowing of the right upper and middle lobes of the lung, as well as blunting of the right costophrenic angle. Blood tests gave the following results: leukocyte count 7.42 x 10^9^/L, neutrophil count 6.53 x 10^9^/L, platelet count 287 x 10^9^/L, hemoglobin level 8.6 g/dL, prothrombin time 15.3 s, and activated partial thromboplastin time 28.7 s. She was experiencing acute renal failure with a serum creatinine level of 783 µmol/L. Liver biochemistry was abnormal with the following values: alkaline phosphatase 513 U/L, alanine aminotransferase 38 U/L, and aspartate aminotransferase 65 U/L. Serum bilirubin level was within the normal range.

The patient was prescribed intravenous ceftriaxone and azithromycin, and hemodialysis was initiated. Within 6 hours of hospitalization, the patient became hypotensive and hypoxemic and required inotropic support and mechanical ventilation. Intravenous ceftazidime and high-dose cloxacillin were substituted for ceftriaxone at that time. Blood cultures obtained on admission were sterile, but penicillin-resistant *S. aureus* grew from cultures of aspirated endotracheal tube secretions. Results of immunofluorescent tests conducted on bronchial washings for viral antigens of influenza virus A and B, parainfluenza virus, respiratory syncytial virus, and adenovirus were negative. Computed tomographic scan of the thorax on day 3 of hospitalization showed widespread confluent consolidation of the right lung with right pleural effusion and patchy consolidation of the lingular lobe of the left lung. The total leukocyte count increased to 26.3 x 10^9^/L, and disseminated intravascular coagulopathy developed. Results of repeated blood and endotracheal cultures were positive for *S. aureus*, and intravenous gentamicin and rifampicin were added to her antimicrobial cocktail. A transthoracic echocardiogram showed a normal heart with no evidence of endocarditis.

Despite aggressive support, the patient's condition continued to deteriorate. A hemopyopneumothorax developed on the right side on day 4 of hospitalization, which required chest tube insertion. Hemoptysis persisted, and inotropic and ventilatory requirements progressively increased. The patient died on day 20 of hospitalization.

The severity of the patient's infection and the clinical symptoms suggested the presence of Panton-Valentine leukocidin genes in the causative *S. aureus*; tests confirmed the suspicion. *S. aureus* was identified on the basis of colony morphologic characteristics, the coagulation of citrated rabbit plasma (bioMérieux, Marcy l'Etoile, France), and production of a clumping factor (Staphyslide test; bioMérieux). DNA was extracted from cultures grown on agar plates and amplified following a previously described protocol (1). The following oligonucleotide primer sequences were used: *luk-PV1*, 5´-ATCATTAGGTAAAATGTCTGGACATGATCCA-3´; *luk-PV2*, 5´-GCATCAAGTGTATTGGATAGCAAAAGC-3´. Polymerase chain reaction products were sequenced commercially and submitted to GenBank (accession no. AY508231).

This case is the first in Singapore of community-acquired pneumonia caused by *S. aureus* in which an attempt was made to detect Panton-Valentine leukocidin genes. Given that the patient had not traveled, she likely acquired the lethal strain of Panton-Valentine leukocidin–positive *S. aureus* locally. This idea is further supported by a recent study which reported that the Panton-Valentine leukocidin gene is found worldwide, albeit in community-acquired strains of methicillin-resistant *S. aureus* (3).

The incidence of severe community-acquired pneumonia attributable to Panton-Valentine leukocidin–positive *S. aureus* is unknown in many parts of the world. With one exception (4), cases of Panton-Valentine leukocidin–positive *S. aureus* causing community-acquired pneumonia have been reported sporadically only from European countries and the United States (1,2,5–8). These results may be attributable to the lack of recognition rather than to the rarity of the condition. A previous report showed that 7.6% of cases of severe community-acquired pneumonia in patients requiring ventilatory support in Singapore were caused by *S. aureus* (9), and a large proportion of these would fit the clinical syndrome described by Gillet et al. (2). Given the ease of transmitting the infection to close contacts (7,10), with the real possibility of a consequent outbreak (10), Panton-Valentine leukocidin testing should be conducted on *S. aureus* strains isolated from all patients with community-acquired necrotizing pneumonia and furunculosis for infection control purposes. Implementing standard hospital methicillin-resistant *S. aureus* measures resulted in control of the outbreak described by Boubaker et al. (10). This measure seems especially relevant given the dismal prognosis offered by conventional therapy in which the death rate of patients with necrotizing pneumonia may reach 75% (2). Further research on the epidemiology, optimal therapy, and prevention of this infection is needed.

## References

[1] Lina G, Piémont Y, Godail-Gamot F, Bes M, Peter MO, Gauduchon V, Involvement of Panton-Valentine leukocidin–producing *Staphylococcus aureus* in primary skin infections and pneumonia. Clin Infect Dis. 1999;29:1128–32. 10.1086/313461 10524952

[2] Gillet Y, Issartel B, Vanhems P, Fournet JC, Lina G, Bes M, Association between *Staphylococcus aureus* strains carrying gene for Panton-Valentine leukocidin and highly lethal necrotising pneumonia in young immunocompetent patients. Lancet. 2002;359:753–9. 10.1016/S0140-6736(02)07877-7 11888586

[3] Vandenesch F, Naimi T, Enright MC, Lina G, Nimmo GR, Heffernan H, Community-acquired methicillin-resistant *Staphylococcus aureus* carrying Panton-Valentine leukocidin genes: worldwide emergence. Emerg Infect Dis. 2003;9:978–84. 12967497 10.3201/eid0908.030089 PMC3020611

[4] Miyashita T, Shimamoto Y, Nishiya H, Koshibu Y, Sugiyama H, Ono Y, Destructive pulmonary embolism in a patient with community-acquired staphylococcal bacteremia. J Infect Chemother. 2002;8:99–102. 11957128 10.1007/s101560200014

[5] Boussard V, Parrot A, Mayaud C, Wislez M, Antoine M, Picard C, Life-threatening hemoptysis in adults with community-acquired pneumonia due to Panton-Valentine leukocidin–secreting *Staphylococcus aureus*. Intensive Care Med. 2003;29:1840–3. 10.1007/s00134-003-1918-5 12904849 PMC7095030

[6] Klein JL, Petrovic Z, Treacher D, Edgeworth J. Severe community-acquired pneumonia caused by Panton-Valentine leukocidin–positive *Staphylococcus aureus*: first reported case in the United Kingdom. Intensive Care Med. 2003;29:1399. 10.1007/s00134-003-1844-6 12783162 PMC7095159

[7] Osterlund A, Kahlmeter G, Bieber L, Runehagen A, Breider JM. Intrafamilial spread of highly virulent *Staphylococcus aureus* strains carrying the gene for Panton-Valentine leukocidin. Scand J Infect Dis. 2002;34:763–4. 10.1080/00365540260348554 12477329

[8] Naimi TS, LeDell KH, Como-Sabetti K, Borchardt SM, Boxrud DJ, Etienne J, Comparison of community- and health care-associated methicillin-resistant *Staphylococcus aureus* infection. JAMA. 2003;290:2976–84. 10.1001/jama.290.22.2976 14665659

[9] Tan YK, Khoo KL, Chin SP, Ong YY. Aetiology and outcome of severe community-acquired pneumonia in Singapore. Eur Respir J. 1998;12:113–5. 10.1183/09031936.98.12010113 9701424

[10] Boubaker K, Diebold P, Blanc DS, Vandenesch F, Praz G, Dupuis G, Panton-Valentine leukocidin and staphylococcal skin infections in schoolchildren. Emerg Infect Dis. 2004;10:121–4. 15078606 10.3201/eid1001.030144 PMC3322757

